# *Solobacterium moorei* promotes the progression of adenomatous polyps by causing inflammation and disrupting the intestinal barrier

**DOI:** 10.1186/s12967-024-04977-3

**Published:** 2024-02-17

**Authors:** Shoujuan Yu, Xifan Wang, Ziyang Li, Dekui Jin, Mengyang Yu, Jingnan Li, Yixuan Li, Xiaoxue Liu, Qi Zhang, Yinghua Liu, Rong Liu, Xiaoyu Wang, Bing Fang, Chengying Zhang, Ran Wang, Fazheng Ren

**Affiliations:** 1 College of Food Science and Nutritional Engineering, China Agricultural University, Beijing, 100083 China; 2 Department of Obstetrics and Gynecology, Columbia University, New York, NY 10032 USA; 3 Key Laboratory of Functional Dairy, Co-Constructed By Ministry of Education and Beijing Government, Department of Nutrition and Health, China Agricultural University, Beijing, 100190 China; 4 Department of General Practice, The Third Centers of Chinese PLA General Hospital, Beijing, 100039 China; 5 Department of Gastroenterology, Peking Union Medical College Hospital, Beijing, 100730 China; 6 Department of Nutrition, The First Center of Chinese PLA General Hospital, Beijing, 100037 China

**Keywords:** Adenomatous polyps, *Solobacterium moorei*, Inflammation, Intestinal barrier

## Abstract

**Background:**

Adenomatous polyps (APs) with inflammation are risk factors for colorectal cancer. However, the role of inflammation-related gut microbiota in promoting the progression of APs is unknown.

**Methods:**

Sequencing of the 16S rRNA gene was conducted to identify characteristic bacteria in AP tissues and normal mucosa. Then, the roles of inflammation-related bacteria were clarified by Spearman correlation analysis. Furthermore, colorectal HT-29 cells, normal colon NCM460 cells, and azoxymethane-treated mice were used to investigate the effects of the characteristic bacteria on progression of APs.

**Results:**

The expression levels of inflammation-related markers (diamine oxidase, d-lactate, C-reactive protein, tumor necrosis factor-α, interleukin-6 and interleukin-1β) were increased, whereas the expression levels of anti-inflammatory factors (interleukin-4 and interleukin-10) were significantly decreased in AP patients as compared to healthy controls. *Solobacterium moorei* (*S. moorei*) was enriched in AP tissues and fecal samples, and significantly positively correlated with serum inflammation-related markers. In vitro, *S. moorei* preferentially attached to HT-29 cells and stimulated cell proliferation and production of pro-inflammatory factors. In vivo, the incidence of intestinal dysplasia was significantly increased in the *S. moorei* group. Gavage of mice with *S. moorei* upregulated production of pro-inflammatory factors, suppressed proliferation of CD4^+^ and CD8^+^cells, and disrupted the integrity of the intestinal barrier, thereby accelerating progression of APs.

**Conclusions:**

*S. moorei* accelerated the progression of AP in mice via activation of the NF-κB signaling pathway, chronic low-grade inflammation, and intestinal barrier disruption. Targeted reduction of *S. moorei* presents a potential strategy to prevent the progression of APs.

**Supplementary Information:**

The online version contains supplementary material available at 10.1186/s12967-024-04977-3.

## Introduction

Colorectal polyps are precancerous lesions of the intestinal epithelium that can progress to colorectal cancer (CRC) and are mainly categorized as serrated polyps or adenomatous polyps (APs), which have been associated with 85% of all cancers of the colon and rectum [1–3]. The recurrence rate of colorectal polyps is reportedly 20–50% [4]. Other than regular colonoscopy and removal, there are currently no effective therapeutic drugs, thus early prevention is essential to reduce the risk of progression from APs to CRC.

The risk of APs is associated with various lifestyle, genetic, and environmental factors. Notably, unhealthy lifestyle habits, such as smoking, alcohol consumption, and regular consumption of high-fat, high-protein diets can increase the prevalence of APs [5]. Each of these factors is associated with compositional and functional changes to the gut microbiota [6]. The initiation and progression of CRC are related to the integrity of the intestinal barrier and immune response elicited by dysbiosis of the gut microbiota [7]. For example, loss of gut barrier function due to enterotoxigenic bacteria, such as *Bacteroides fragilis*, can induce activation of STAT3 and NF-κB, which promote inflammation [8], enhance epithelial permeability, and contribute to bacterial invasion of the intestinal epithelium, ultimately leading to the formation of APs and development of CRC [9].

Through improvements to analytical technologies, it has become evident that changes to the gut microbiome are associated with an increased risk of APs [10, 11]. For example, Shen et al. found that the proportion of Proteobacteria was enriched in the gut microbiota of adenoma patients, while Bacteroidetes were enriched in healthy individuals [12, 13]. In contrast, Chen et al. found that the abundances of Bacteroidetes, *Enterococcus*, and *Streptococcus* species were increased in the gut of adenoma patients [14]. In addition, Lu et al. reported that greater amounts of Firmicutes, Proteobacteria, Bacteroidetes, and Actinobacteria were associated with APs as compared to normal mucosa [15]. However, Magnifesta et al. found no overall changes at the phylum level in APs as compared to adjacent healthy mucosa [16]. Nonetheless, at present, there is no uniform consensus on the types of bacteria associated with the development of APs. Considering the potential implications of APs, identification of the characteristic bacteria would be useful for diagnosis and possible therapeutic interventions.

In this study, comparisons of the differential bacteria in AP tissues and normal mucosa were conducted to identify the characteristic bacteria of AP tissues. The roles of the characteristic bacteria in the progression of APs to CRC were explored both in vitro and in vivo. In vitro studies were conducted to explore the effects of characteristic bacteria on the proliferation of colorectal cells and production of inflammatory factor. In addition, in vivo studies were performed to investigate the effects of characteristic bacteria on the expression profiles of inflammatory factors, alterations to the proportions of immune cells, and the ultimate effects on the intestinal barrier. The results of this study provide useful references for subsequent research on the relationship between characteristic bacteria and progression of APs to CRC.

## Materials and methods

### Cohort recruitment and sample collection

Ethical approval was granted by the Human Research Ethics Committee of China Agricultural University (CAUHR-2021020). Prior to inclusion in this study, written informed consent was obtained from all subjects. Tissue specimens were obtained from 39 AP patients and 39 healthy individuals who underwent colonoscopy at The Third Medical Center of PLA General Hospital (Beijing, China). The inclusion criteria were age of 18–60 years and confirmation of low-grade APs by colonoscopy and pathological analysis, while the exclusion criteria were a history of inflammatory bowel disease, irritable bowel syndrome, diabetes, hypertension, any acute or chronic coexisting illness, and neoadjuvant chemotherapy or antibiotic treatment one month prior to surgery. All patients received macrogol laxatives preoperatively. AP tissues and adjacent normal mucosa located about 10 cm away from the lesion were obtained from each patient and collected in cryogenic vials under strict aseptic conditions.

Blood samples were collected from healthy controls and AP patients and centrifuged at 3000 × *g* for 15 min to obtain serum [17]. Commercial kits were used to measure diamine oxidase (DAO) activity (Nanjing Jiancheng Bioengineering Institute, Nanjing, China) and serum levels of D-lactate (D-LA) (Beijing Solarbio Science & Technology Co., Ltd., Beijing, China). Enzyme-linked immunosorbent assay (ELISA) kits (Hangzhou MultiSciences (Lianke) Biotech, Co., Ltd., Hangzhou, China) were used to measure serum levels of C-reactive protein (CRP), tumor necrosis factor-α (TNF-α), interleukin (IL)-6, IL-1β, IL-4, and IL-10. All assays were conducted in accordance with the manufacturers’ instructions.

### Sequencing and analysis of the 16S rRNA gene

Sequencing of the 16S rRNA gene was conducted as described elsewhere [7]. The biopsy tissue samples were disinfected by soaking in betadine antiseptic solution for 3 min and washing three times with sterile phosphate-buffered saline (PBS). Then, the surface tissue was removed to eliminate mucosal microbiota and other contaminants. The last wash buffer was analyzed by quantitative real-time polymerase chain reaction (qRT-PCR) analysis and 16S rRNA gene sequencing to confirm the absence of contamination. Sequencing of the 16S rRNA gene was performed by Majorbio Biomedical Technologies Ltd. (Shanghai, China). The raw data were analyzed with an online platform available at https://cloud.majorbio.com [18–21].

### Metagenomic shotgun sequencing

Fecal samples from healthy controls and AP patients were immediately frozen and stored at -80 °C until use. Whole-genome shotgun metagenome sequencing was performed for taxonomic and functional analyses of the gut microbiome. Library preparation and subsequent metagenomic sequencing were conducted with the BGISEQ-500 platform (Beijing Genomics Institute, Beijing, China) with 150-bp paired-end reads at BGI Biotech Co., Ltd. (Shenzhen, China), targeting > 20 Gb of sequencing data per sample. The raw sequencing data were processed using the Trimmomatic v0.36 tool (https://github.com/usadellab/Trimmomatic/releases), which included trimming of the adapters and deletion of low-quality reads or base pairs. Then, the Bowtie 2 tool (https://bowtie-bio.sourceforge.net/bowtie2/index.shtml) was used to remove host contamination by mapping against the reference human genome (version hg38) [22, 23]. Subsequently, clean reads were constructed and further taxonomically profiled using the MetaPhlAn4 computational tool (https://github.com/biobakery/MetaPhlAn) with default parameters.

### Bacterial strains and culture conditions

*Solobacterium moorei* (*S.moorei*) strains (German Collection of Microorganisms and Cell Cultures GmbH, Braunschweig, Germany) were maintained in medium 104 supplemented with hemin and vitamin K1 in an anaerobic jar (80% N_2_, 10% H_2_, 10% CO_2_) at 37 °C. *Escherichia coli* MG1655 (*E.coli*) strain (American Type Culture Collection, Manassas, VA, USA) was cultured in lysogeny broth at 37 °C.

### Cell culture

Colon cancer (HT-29) cells (American Type Culture Collection) and normal colon immortalized epithelial (NCM460) cells (Incell Corporation LLC, San Antonio, TX, USA) were grown in Roswell Park Memorial Institute 1640 medium (Gibco, Carlsbad, CA, USA) and Dulbecco’s modified Eagle’s medium (Gibco), respectively, supplemented with 10% fetal bovine serum (Gibco) and 1% penicillin–streptomycin at 37 °C under an atmosphere of 5% CO_2_/95% air.

### Bacterial attachment assay

Colon cells (1 × 10^5^) were grown in the wells of a 24-well plate and co-cultured with bacteria for 2 h (multiplicity of infection (MOI) = 10) under anaerobic conditions. Afterward, the medium was removed and the cells were washed three times with PBS, lysed by the addition of 100 μL of ultrapure water for 20 min, and then homogenized by the addition 400 μL of medium. The attached *S. moorei* colonies were recovered on enhanced clostridium medium plates under anaerobic conditions and quantified. *E. coli* was used as a negative control.

### Cell proliferation capacity assay

Cell proliferation capacity was measured with the Cell Counting Kit-8 (Dojindo Laboratories Co., Ltd., Kumamoto, Japan). In brief, 1 × 10^4^ colon cells were grown in the wells of a 24-well plate and co-cultured with bacteria (MOI = 100) for 2 h under anaerobic conditions. Afterward, the medium was replaced with fresh medium (10% FCS, 1% penicillin–streptomycin, and 20 μg/mL of gentamycin were added). The proliferation capacity of the cells was assayed at 0, 24, 48, and 72 h after the addition of WST-8 solution (2-(2-methoxy-4-nitrophenyl)-3-(4-nitrophenyl)-5-(2,4-disulfophenyl)-2H-tetrazolium sodium salt) to the medium and incubation at 37 °C for 2 h under an atmosphere of 5% CO_2_/95% air. The optical density of the supernatant at 450 nm was measured with a spectrophotometer.

### Measurement of inflammatory factors in cell supernatant

Colon cells (1 × 10^4^) were grown in the wells of a 24-well plate and co-cultured with bacteria (MOI = 100) for 2 h under anaerobic conditions for 3 days. Afterward, the levels of TNF-α, IL-6, IL-1β, IL-4, and IL-10 in the supernatant were measured with ELISA kits (Hangzhou MultiSciences (Lianke) Biotech, Co., Ltd.) in accordance with the manufacturer’s instructions.

### Animal experiments

All animal studies were approved by the Animal Experimentation Ethics Committee of China Agricultural University (approval no. AW82303202-5-2). Male C57BL/6 J mice (n = 40; age, 8 weeks; Laboratory Animal Center of Vital River Laboratory Animal Technology Co., Ltd., Beijing, China) were housed in an air-conditioned room in a standard animal care facility at a constant temperature of 22–25 °C and relative humidity of 60% under a 12-h light/dark cycle and fed a standard diet for 1 week. A schematic of the experimental design is provided in Fig. 4A. All mice (excluding the control group) were treated with a cocktail of broad-spectrum antibiotics (ampicillin, 0.2 g/L; vancomycin, 0.1 g/L; neomycin, 0.2 g/L; and metronidazole, 0.2 g/L) in drinking water for 2 weeks before intraperitoneal administration of azoxymethane (AOM) at 10 mg/kg body weight (BW). At 3 days after AOM administration, the mice were gavaged with PBS (AOM group), 1 × 10^8^ colony-forming units (CFU) of *S. moorei* (*S. moorei* + AOM group), or 1 × 10^8^ CFU of *E. coli* (*E. coli* + AOM group) every day for 8 weeks. Afterward, the mice were anesthetized by CO_2_ inhalation and sacrificed by cervical dislocation.

### Fluorescence in situ hybridization (FISH)

FISH was performed as described in previous reports [24, 25]. Colon tissues were washed with PBS, fixed with 10% formalin solution, embedded in paraffin, and cut into 5 μm-thick slices, which were incubated overnight at 50 °C with a fluorescein-conjugated probe against *S. moorei* (5′–CCT TAC AAA CAA GAG CTT TTA CA–3′). A nonspecific probe (5′-ACT CCT ACG GGA GGC AGC-3′) was used as a negative control. Then, the sections were incubated with an antibody (Ab) against mucin 2 (Wuhan Servicebio Technology Co., Ltd., Wuhan, China) at 4 °C for 12 h, followed by goat anti-mouse IgG (H + L) cross-linked with an Alexa fluor™ 568-labelled secondary Ab (Thermo Fisher Scientific, Waltham, MA, USA) at room temperature for 1 h.

### Histopathological analysis

Colon tissues slices were stained with hematoxylin and eosin for histologic diagnosis by an experienced pathologist who was unaware of the treatment allocation of the mice. Inflammation was assessed by infiltration of inflammatory cells into the epithelium, whereas dysplasia was defined by the presence of hyperchromasia, nuclear pleomorphism, increased nuclear-to-cytoplasmic ratios, and atypical mitotic figures [24, 25].

### Immunohistochemical analysis

Paraffin-embedded colon tissues on glass slides were deparaffinized, antigen-retrieved, blocked, and incubated with an Abs against Ki67 (Abcam, Cambridge, UK), cluster of differentiation CD4 (Santa Cruz Biotechnology, Inc., Dallas, TX, USA), and CD8 (Santa Cruz Biotechnology, Inc.) followed by an enzyme-conjugated secondary Ab. Afterward, the slides were counterstained with hematoxylin and the chromogen 3,3'-diaminobenzidine. The proliferation index was determined by the number of Ki67 + cells in each crypt. At least 30 random fields of six sections were analyzed for each tissue specimen (n = 6).

## Intestinal permeability assay

Intestinal permeability was evaluated using a fluorescein isothiocyanate (FITC)-dextran probe (4 kDa, Sigma-Aldrich Corporation, St. Louis, MO, USA). After fasting for 4 h, the mice were gavaged with the FITC-dextran probe (600 mg/kg BW). At 4 h after gavage, blood was collected from the orbital venous sinus and centrifuged at 3000 × *g* for 15 min to collect serum, which was analyzed using a fluorescence spectrometer (excitation, 485 nm; emission, and 535 nm). A standard curve of the FITC-dextran probe diluted in PBS was generated. DAO activities and D-LA concentrations were measured with commercial kits as described above.

### Alcian blue-periodic acid Schiff staining

Colon tissues were collected, fixed with Carnoy's fixative solution for no more than 12 h, dehydrated, embedded in paraffin, and cut into sections, which were stained with Alcian blue-periodic acid Schiff (Beijing Solarbio Science & Technology Co., Ltd.). The thickness of the mucosal layer and number of goblet cells in at least 30 random fields of six sections for each sample (n = 6) were determined with ImageJ software (https://imagej.net/ij/).

### Western blot analysis

Western blot analysis was conducted to determine the expression levels of proteins associated with APs. Total proteins were isolated from tissues using radio immunoprecipitation assay (RIPA) lysis buffer containing 1% phenylmethane sulfonyl fluoride (Beyotime Institute of Biotechnology, Shanghai, China) and quantified using a bicinchoninic acid assay kit (Beijing Solarbio Science & Technology Co., Ltd.). Equal concentrations of proteins were separated by electrophoresis and then transferred to polyvinylidene fluoride membranes (EMD Millipore Corporation, Billerica, MA, USA), which were probed with primary and secondary Abs against ZO1 (Cat# 21773-1-AP, RRID: AB_10733242), Occludin (Cat# GB111401, RRID: AB_2880820), Claudin-1 (Cat# 28674-1-AP, RRID: AB_2881190), β-actin(Cat# 20536-1-AP, RRID: AB_10700003), tubulin(Cat# 11224-1-AP, RRID: AB_2210206) (all, Proteintech, Rosemont, IL, USA),β-Catenin(Cat# ab32572, RRID: AB_725966), p-Gsk3β (Cat# ab75745, RRID: AB_1310290), cyclin D1 (Cat# ab134175, RRID: AB_2750906), E-cadherin (Cat# ab231303, RRID:AB_2923285), NF-kB p65 (Cat# ab16502, RRID:AB_443394), NF-kB p65 (phospho S536) (Cat# ab76302, RRID:AB_1524028), IκB alpha antibody (Cat# ab32518, AB_733068) and IκB alpha (phospho S36) (Cat# ab133462, AB_2801653) (all, Abcam). The protein bands were visualized with enhanced chemiluminescence reagent (Beijing Solarbio Science & Technology Co., Ltd.) and quantified with the Amersham™ Imager 600 System (GE HealthCare, Chicago, IL, USA).

### Measurement of inflammatory factor concentrations in serum and colon tissues

The concentrations of inflammatory cytokines (TNF-α, IL-6, IL-1β, IL-4, and IL-10) in serum and colon tissues were measured using ELISA kits (Hangzhou MultiSciences (Lianke) Biotech, Co., Ltd.) in accordance with the manufacturer’s instructions. Serum was collected by centrifugation of whole blood at 3000 × *g* for 10 min at 4 °C. Colon tissues were homogenized in RIPA buffer (Beyotime Institute of Biotechnology) supplemented with a protease inhibitor cocktail (Beyotime Institute of Biotechnology) and then centrifuged at 12,000 × *g* for 30 min at 4 °C. The optical density of the supernatant was measured at 570 nm.

### qRT-PCR analysis

The gene expression levels of pro-inflammatory cytokines (TNF-α, IL-6, and IL-1β) and tight junction proteins (ZO1, occludin, and claudin-1) in the mouse colon were quantified by qRT-PCR. In brief, total RNA was isolated with RNA extraction kit (Qiagen GmbH, Hilden Germany) and reverse transcribed into complementary DNA using All-In-One 5X RT MasterMix (Applied Biological Materials Inc., Vancouver, BC, Canada) and the primers listed in Table 1. The amount of *S. moorei* in mouse fecal samples was quantified by qRT-PCR. In brief, *S*. *moorei* DNA was extracted from mouse fecal sample using the TIANamp Bacteria DNA Kit (Tiangen Biotech (Beijing) Co., Ltd., Beijing, China) in accordance with the manufacturer’s instructions. The amount of *S. moorei* in the mouse fecal samples was determined in reference to a standard curve.

**Table 1:** Primer sequences of RT-PCR

Gene Name	Forward 5′-3′	Reverse 5′-3′
TNF-α	CCCTCACACTCAGATCATCTTCT	GCTACGACGTGGGCTACAG
IL-6	TAGTCCTTCCTACCCCAATTTCC	TTGGTCCTTAGCCACTCCTTC
IL-1β	GAAATGCCACCTTTTGACAGTG	TGGATGCTCTCATCAGGACAG
GAPDH	CCGAGAATGGGAAGCTTGTC	TTCTCGTGGTTCACACCCATC
ZO1	GCCGCTAAGAGCACAGCAA	TCCCCACTCTGAAAATGAGGA
Occludin	TTGAAAGTCCACCTCCTTACAGA	CCGGATAAAAAGAGTACGCTGG
Claudin-1	TGCCCCAGTGGAAGATTTACT	CTTTGCGAAACGCAGGACAT

### Measurement of hydrogen sulfide (H_2_S) concentrations in colon tissues

The amount of H_2_S in mouse colon tissues was quantified using a colorimetric assay kit (Elabscience Bionovation Inc., Houston, TX, USA) in accordance with the manufacturer’s instructions.

### Statistical analysis

All statistical analyses were performed using IBM SPSS Statistics for Windows, version 25.0. (IBM Corporation, Armonk, NY, USA) and Prism 8 software (GraphPad Software, Inc., San Diego, CA, USA). The student’s *t*-test was used for comparisons of two groups and one-way analysis of variance (ANOVA) for comparisons of three or more groups. A probability (*p*) value < 0.05 was considered statistically significant. The data are presented as the mean ± standard deviation (SD).

## Results

### Description of the study cohort

The characteristics of the 39 AP patients and 39 healthy controls (age, sex, body mass index [BMI], alcohol use, smoking status, and disease history) are shown in Table 2. The mean age of the AP patients and healthy controls was 54 ± 9.8 and 53 ± 9.6 years, the percentage of males was 79.5% and 76.9%, and the mean BMI was 25.7 ± 3.0 and 25.4 ± 3.0, respectively. A BMI ≥ 25 was considered overweight. About 50% of the AP patients and healthy controls consumed alcohol and about 70% were non-smokers. There were also no reports of antibiotic or probiotic use in the last month. The proportions of APs observed on the right and left side of the colon were 41% and 59%, respectively.

**Table 2:** Patient characteristics

	AP patients (n = 39)	Healthy controls (n = 39)
Age (mean, ± SD)	54 ± 9.8	53 ± 9.6
Sex (n, males, %)	31 [79.5]	30 [76.9]
BMI	25.7 ± 3.0	25.4 ± 3.0
Alcohol intake status (n, %)		
Active	20 [51.3]	19 [48.7]
Quit	10 [25.6]	9 [23.1]
Never	9 [23.1]	11 [28.2]
Smoking status (n, %)		
Active	11 [28.2]	10 [25.6]
Quit	2 [5.1]	2 [5.1]
Never	26 [66.7]	27 [69.2]
Probiotic use	0 [0]	0 [0]
Antibiotic exposure	0 [0]	0 [0]
Localization of polyps (n, %)		
Cecum (right)	1 [2.6]	/
Colon Ascendes (right)	5 [12.8]	/
Colon Transversum (right)	10 [25.6]	/
Colon Descendens (left)	6 [15.4]	/
Sigmoid (left)	15 [38.5]	/
Rectum (left)	2 [5.1]	/

### Increased intestinal permeability and pro-inflammatory factors in AP patients and healthy controls

**Fig. 1:**
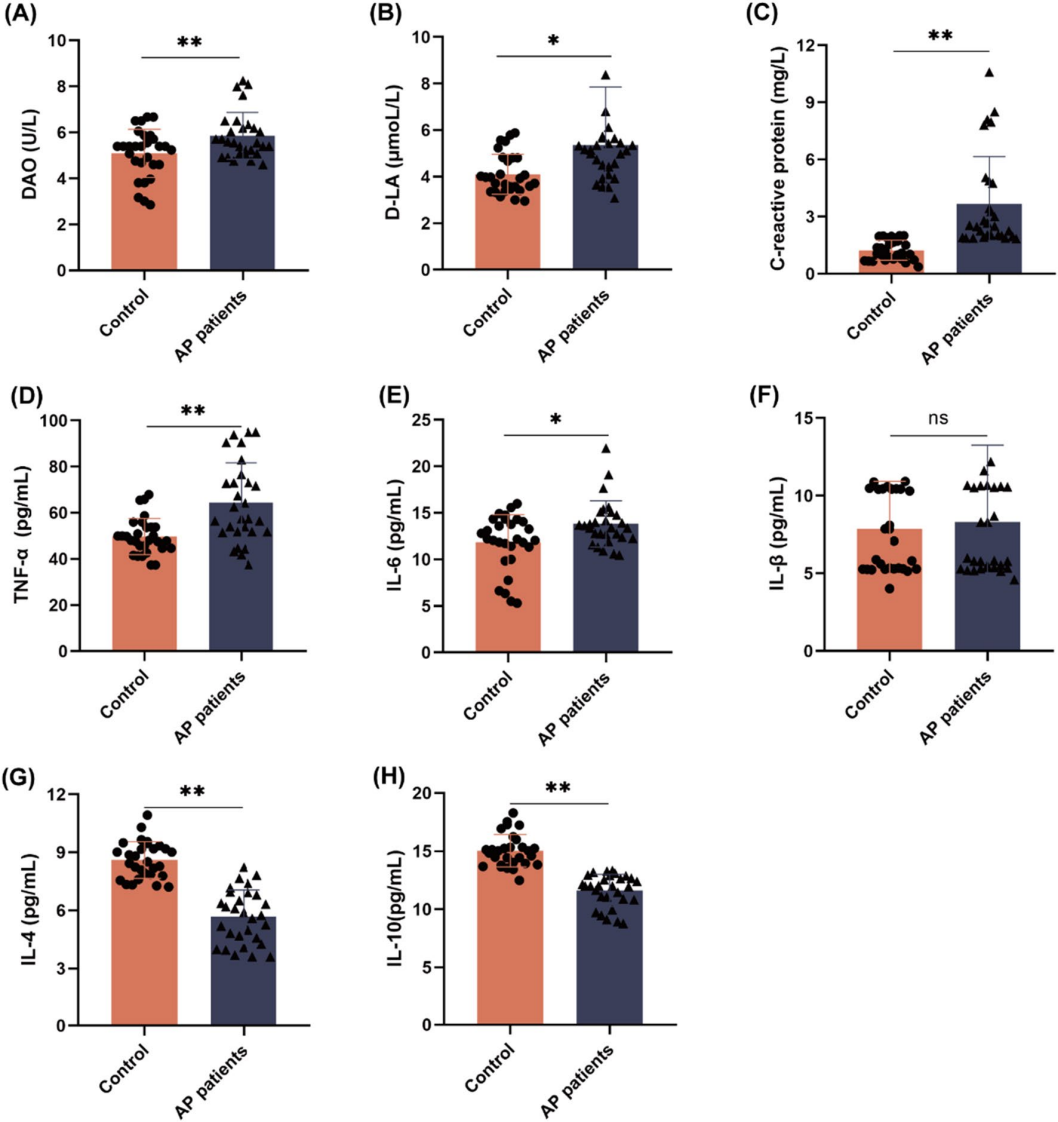
Intestinal permeability and concentrations of inflammatory factors in healthy controls and AP patients. **A** DAO activity in human serum. **B** D-LA concentrations in human serum. **C** CRP concentrations in human serum. **D**–**H** Serum concentrations of TNF-α **D**, IL-6 (E), IL-1β **F**, IL-4 **G**, and IL-10 **H** in the healthy controls and AP patients. The data are expressed as the mean ± SD (n = 29). *p < 0.05 and **p < 0.01 (two-tailed unpaired Student’s *t*-test)

DAO activity and serum concentrations of D-LA and inflammatory factors (CRP, TNF-α, IL-6, IL-1β, IL-4, and IL-10) were measured to assess potential relationships between immune-related markers and the presence of APs. DAO activity was significantly higher in the AP patients compared to healthy controls (5.85 ± 1.02 vs. 5.09 ± 1.05 U/L, respectively, *p* < 0.01, Fig. 1A). Similarly, serum concentrations of D-LA were significantly higher in the AP patients than the healthy controls (5.36 ± 2.54 vs. 4.11 ± 2.54 μmol/L respectively, *p* < 0.05, Fig. 1B). Meanwhile, serum concentrations of the pro-inflammatory factors CRP, TNF-α, IL-6, and IL-1β were higher in the AP patients than the healthy controls by 195.7% (*p* < 0.01), 24.5% (*p* < 0.01), 16.8% (*p* < 0.05), and 5.6% (*p* < 0.05), respectively. Serum concentrations of the anti-inflammatory factors IL-4 and IL-10 were lower in the AP patients than the healthy controls by 34.1% (*p* < 0.01) and 22.7% (*p* < 0.01) respectively (Fig. 1C–H). These results indicate that AP patients had elevated intestinal permeability, increased serum levels of pro-inflammatory factors, and decreased serum levels of anti-inflammatory factors as compared to the healthy controls.

### *S. moorei* was enriched in AP tissues and positively associated with intestinal permeability and inflammation

The microbiota taxonomic profile of the AP tissues and matched normal mucosal specimens was assessed by sequencing of the V3-V4 region of the 16S rRNA gene. Since the amount of some tissue specimens was insufficient for sequencing, only 28 APs and 32 normal mucosal specimens were sequenced. Overall, 572 and 596 unique operational taxonomic units (OTUs) were identified from the AP tissues and normal mucosal specimens, respectively (Additional file 1: Fig. S1).

**Fig. 2:**
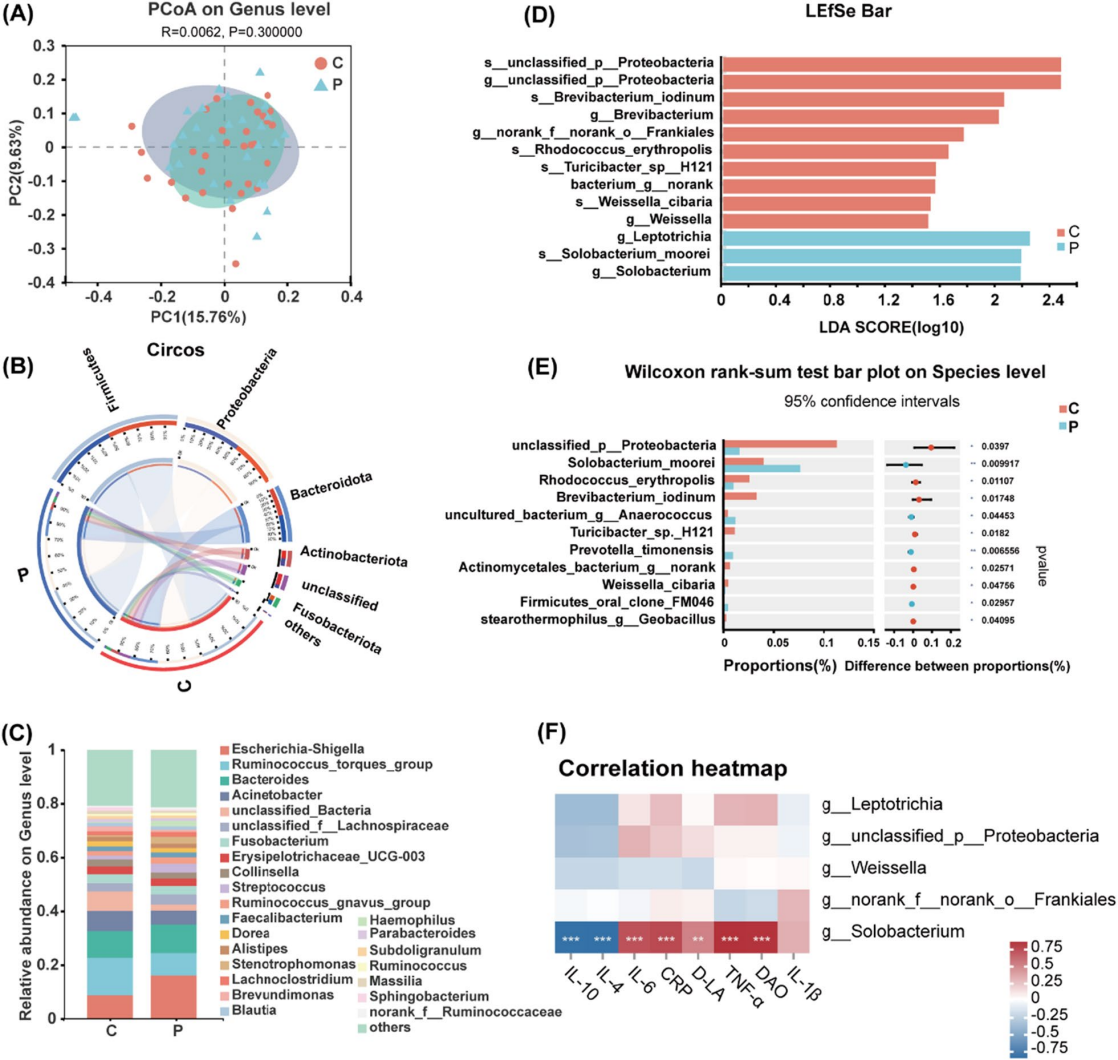
Bacterial composition of normal mucosa and AP tissues. **A** PCoA plot of unweighted Unifrac distances. R^2^ and *p* values were calculated using ANOSIM (Analysis of Similarities). **B** Relative abundances of different phyla in normal mucosa (n = 32) and AP tissues(n = 28). **C** Relative abundances of different genera in normal mucosa and AP tissues. **D** LEfSe analysis. The only criterion for feature selection was log LDA score > 1.5. **E** Wilcoxon rank-sum test comparing the flora characteristics of normal mucosa and AP tissues. **F** Correlation between serum inflammatory factors and signature microbiota. *p < 0.05, **p < 0.01, and ***p < 0.001 (Spearman correlation test). C, normal mucosa; P, AP tissues

The alpha diversity index revealed that the normal mucosal specimens had greater bacterial diversity and richness as compared to the AP tissues, although these differences were not statistically significant (Additional file 1: Table S1). Principal coordinates analysis (PCoA) based on the relative abundance of OTUs revealed a slight separation of the normal mucosa and AP tissues based on the first two principal component scores, which accounted for 15.76% and 9.63% of the total variations (Fig. 2A), suggesting that the presence of APs may have caused changes to the structure of the gut microbiota.

The most common phyla of the two groups included Firmicutes, Proteobacteria, Bacteroidetes, Actinobacteria, and Fusobacteriota (Fig. 2B). At the genus level, the dominant bacteria identified in the APs and normal mucosal specimens were *Escherichia-Shigella, Ruminococcus_torques_group, Bacteroides*, and *Actinobacter* (Fig. 2C). LEfSe (linear discriminant analysis effect size) analysis was performed to identify the characteristic bacteria of the AP tissues and histograms of the LDA scores (> 1.5) were generated to determine the differential bacterial taxa in the APs and normal mucosal specimens. The results showed that pathogenic bacteria (e.g., *Leptotrichia* and *Solobacterium*) were enriched in AP tissues (*p* < 0.05), while the probiotics *Weissella* (*p* = 0.048) and *Brevibacterium* (*p* = 0.005) were enriched in healthy control tissues (Fig. 2D and Additional file 1: Fig. S2).

The results of the Wilcoxon test (Fig. 2E) revealed that *S. moorei* was significantly increased in the AP tissues by 178% as compared to the normal mucosal specimens (*p* = 0.010). The Spearman correlation test was used to explore the relationship between characteristic bacteria in AP tissues and inflammation-related markers. As shown in Fig. 2F, only *S. moorei* was significantly and positively correlated with the inflammation-related markers, especially DAO activity (R^2^ > 0.8, *p* < 0.01) and serum TNF-α (R^2^ > 0.8, *p* < 0.01). In addition, *S. moorei* was significantly negatively correlated with the anti-inflammatory factors IL-4 and IL-10 (R^2^ < -0.8, *p* < 0.01). These results suggest that *S. moorei* was associated with increased intestinal permeability as well as pro-inflammatory factors.

In addition, the characteristics of bacterial flora in fecal samples from both healthy controls and AP patients were examined (Additional file 1: Fig. S3). As shown in Additional file 1: Fig. S3A, the results of PCoA showed that there was no significant difference in β diversity at the genus level between the AP patients and healthy controls (*p* = 0.4). The most common phyla in the fecal samples were Bacteroidetes, Firmicutes, Proteobacteria, Actinobacteria, and Fusobacteriota (Additional file 1: Fig. S3B), while the dominant genera were *Phocaeicola, Bacteroides, Prevotella*, and *Faecalibacterium* (Additional file 1: Fig. S3C). LEfSe analysis revealed that *Blautia*, *Anaerotignum*, and *Anaerobutyricum* were enriched in the fecal samples of the healthy controls, while *Megasphaora*, *Clostridiaceae_unclassified*, *Rikenellaceae_unclassified*, and *Solobacterium* were enriched in the fecal samples of the AP patients (Additional file 1: Fig. S3D). In summary, *S. moorei* was enriched in both tissues and feces of AP patients, and *S. moorei* showed a positive correlation with pro-inflammatory factors, implying that *S. moorei* may have the potential to promote the development of APs.

### *S. moorei* preferentially induces proliferation of colorectal cells and promotes inflammation

**Fig. 3:**
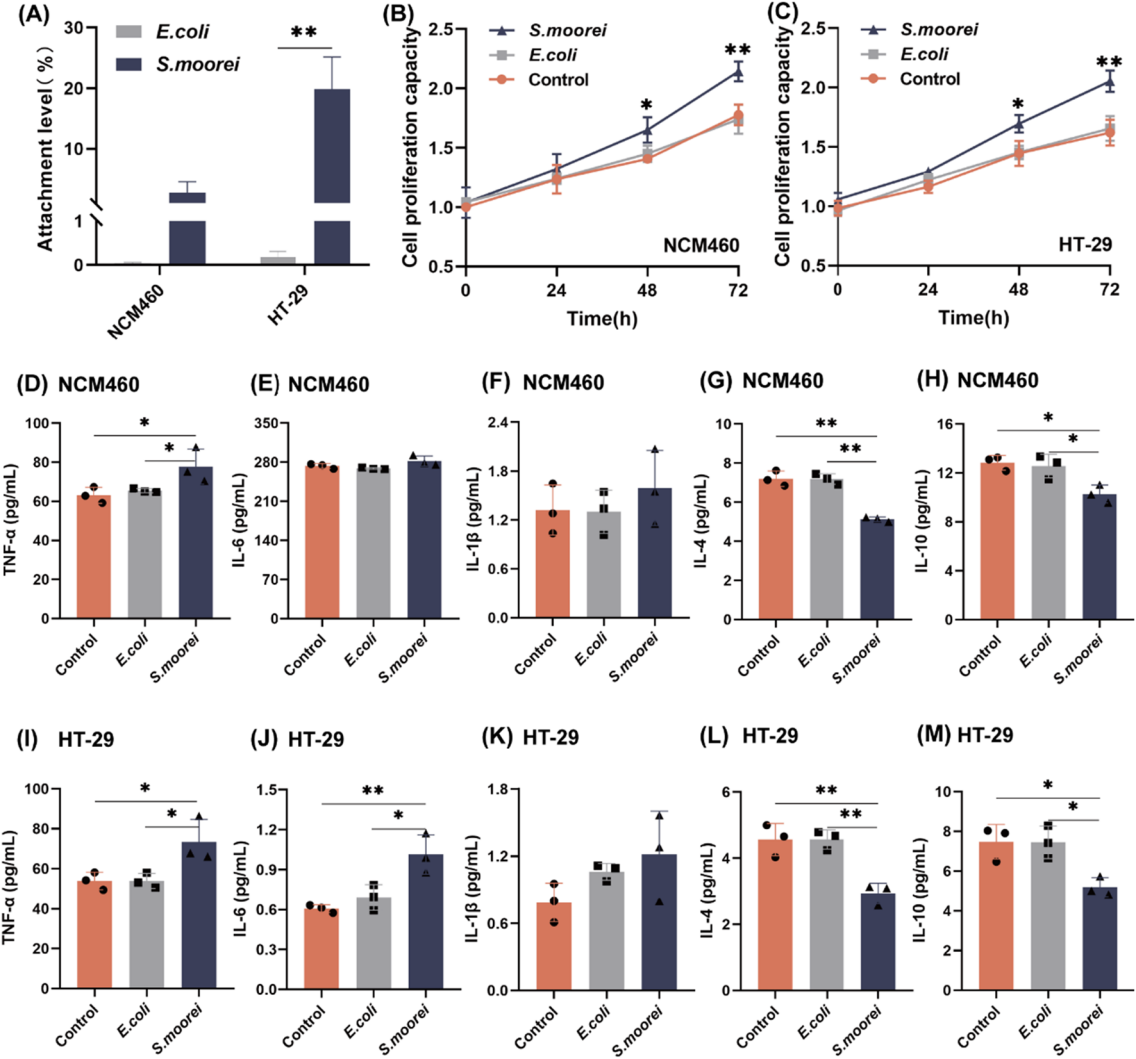
Effects of *S. moorei* on proliferation and inflammation of NCM460 and HT-29 cells. **A** Level of attachment of *S. moorei* to colon epithelial NCM460 cells and colon cancer HT-29 cells (MOI = 10 for 2 h). *E. coli* was used as a negative control. **B** Proliferation capacity of NCM460 cells after *S. moorei* treatment. *E. coli* was used as a negative control. **C** Proliferation capacity of HT-29 cells after *S. moorei* treatment. *E. coli* was used as a negative control. **D**–**H** Concentrations of TNF-α (**D**), IL-6 (**E**), IL-1β (**F**), IL-4 **G** and IL-10 **H** in the supernatant of NCM460 cells after *S. moorei* treatment. **I**–**M** Concentrations of TNF-α (**G**), IL-6 (**H**), and IL-1β (**I**), IL-4 (**G**) and IL-10 (**H**) in the supernatant of HT-29 cells after *S. moorei* treatment. Data are presented as the mean ± SD of three biological replicates (n = 3). *p < 0.05 and **p < 0.01 (one-way ANOVA)

The attachment properties of *S. moorei* were investigated in vitro using CRC HT-29 cells and normal colon mucosal epithelial NCM460 cells. The bacterial attachment assay revealed that *S. moorei* attached more readily to NCM460 and HT-29 cells than *E. coli* (Fig. 3A). Notably, *S. moorei* preferred attachment to HT-29 cells (*p* < 0.01).

Next, *S*. *moorei* and *E*. *coli* were each co-cultured with colon cells to assess cell proliferation. The cells were co-cultured with *S. moorei* or *E. coli* (MOI = 100) for 2 h/day under anaerobic conditions for 3 days. As shown in Fig. 3B–C, after 48 h of co-culture, *S. moorei* significantly increased proliferation of both NCM460 and HT-29 cells (both, *p* < 0.05) as compared to the controls. Similar results were obtained after 72 h of co-culture. However, there was no significant difference in proliferation of colorectal cells co-cultured with *E. coli* and the control group.

Finally, concentrations of the inflammatory factors were quantified in the supernatants after co-culture of *S. moorei* or *E. coli* with colorectal cells. As shown in Fig. 3D–M, TNF-α concentrations were significantly higher in the supernatants of NCM460 and HT-29 cells with *S. moorei* than the control and *E. coli* groups (both, *p* < 0.05). Likewise, the IL-6 concentration in the supernatant of HT-29 cells co-cultured with *S. moorei* was significantly higher than the control and *E. coli* groups (*p* < 0.01 and < 0.05, respectively). There was no significant difference in the concentration of IL-1β between the two groups. Notably, the concentrations of the anti-inflammatory factors IL-4 and IL-10 were significantly decreased in the *S. moorei* group as compared to the control NCM460 and HT-29 cell groups (both, *p* < 0.05). Collectively, these results indicate that *S. moorei* may contribute to the development of APs by stimulating cell proliferation and the production of pro-inflammatory factors.

### *S. moorei* aggravated colon dysplasia in mice

**Fig. 4:**
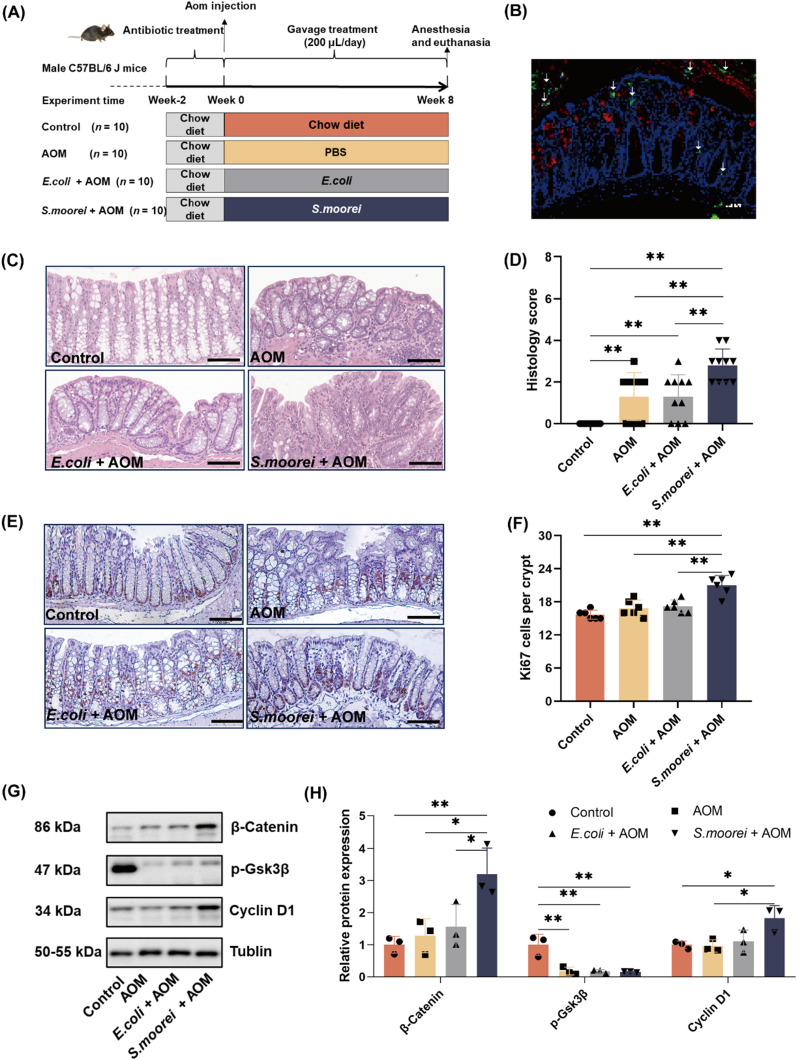
Effect of *S. moorei* on AOM-induced progression of APs in mice. **A** Schematic of the experimental design and timeline of mice models. **B** FISH analysis of colon sections using *S. moorei* probes (green) and mucin2 (red) antibodies. **C** *S. moorei* promoted dysplasia of colon tissues. Representative histologic images of mouse colon tissues stained with hematoxylin and eosin. Scale bars = 100 μm. **D** Histology score indicating degree of inflammation and dysplasia in mice (n = 10). **E** Immunohistochemical analysis of Ki67 + cells in colon tissues. Scale bars = 100 μm. **F** Quantitative analysis of Ki67 + cells (n = 6). **G** Representative western blot images of β-catenin, p-Gsk3β, and cyclin D1 protein expression in the mice colon. **H** Quantification of β-catenin, p-Gsk3β, and cyclin D1 protein levels (n = 3).Data are presented as the mean ± SD. *p < 0.05 and **p < 0.01 (one-way ANOVA)

A mouse model of AOM-induced colorectal lesions was created to determine whether *S. moorei* accelerates the progression of APs. A schematic of the animal experiment was shown in Fig. 4A. In brief, microbiota-depleted mice received a single gavage of *S. moorei* (1 × 10^8^) at 3 days after AOM treatment (10 mg/kg BW), while the negative controls were gavaged with nonpathogenic *E. coli* (1 × 10^8^ CFU) or PBS. The FISH results showed that *S. moorei* colonized the mucosal layer and intestinal epithelial cells of mice with *S. moorei* (green fluorescence indicated by white arrows in Fig. 4B). Only the mucus layer (red) and nucleus (blue) were detected in control, AOM, and* E.coli* groups, and no colonization by *S. moorei* (green) was detected in any of the three groups (Additional file 1: Fig. S4). The results of qRT-PCR analysis confirmed that *S. moorei* colonized the mouse colon after 8 weeks of gavage (Additional file 1: Fig. S5).

Finally, histological analysis of the whole intestinal tract of each mouse was conducted to assess the degree of damage among the treatment groups. As shown in Fig. 4C, the colon tissues of mice in the AOM group exhibited hyperchromasia, mild heterogeneity of the intestinal mucosal glands (with enlarged lumens), mild atypical hyperplasia, and inflammation, similar to the mice exposed to *E. coli*. However, the colon tissues of mice exposed to *S. moorei* showed intestinal crypt loss, high-grade atypical hyperplasia, and inflammation. As shown in Fig. 4D, Additional file 1: Fig. S6, and Additional file 1: Table S2, the composite histology score was significantly higher in the *S. moorei* group than the AOM and *E. coli* groups (2.70 ± 0.95, 1.30 ± 1.16, and 1.30 ± 1.06, respectively, *p* < 0.01). In addition, the number of Ki67-positive cells, as a marker of cell proliferation, was significantly greater in the *S. moorei* group than the normal control and AOM groups *(p* < 0.01, Fig. 4E, F). Also, the protein expression levels of β-catenin and cyclin D1 (Wnt-related proteins) were significantly upregulated in the *S*. *moorei* group (Fig. 4G, H). While expression of p-Gsk3β (Wnt antagonist) was significantly decreased in the AOM group as compared to the control group, there was no significant difference between the *S*. *moorei* and AOM groups. Notably, accumulation of β-catenin triggers activation of the Wnt signaling pathway. These results suggest that *S. moorei* may promote the development of lesions and abnormal cell proliferation in mice by activating the Wnt signaling pathway.

### *S. moorei* disrupted intestinal barrier function in mice

**Fig. 5:**
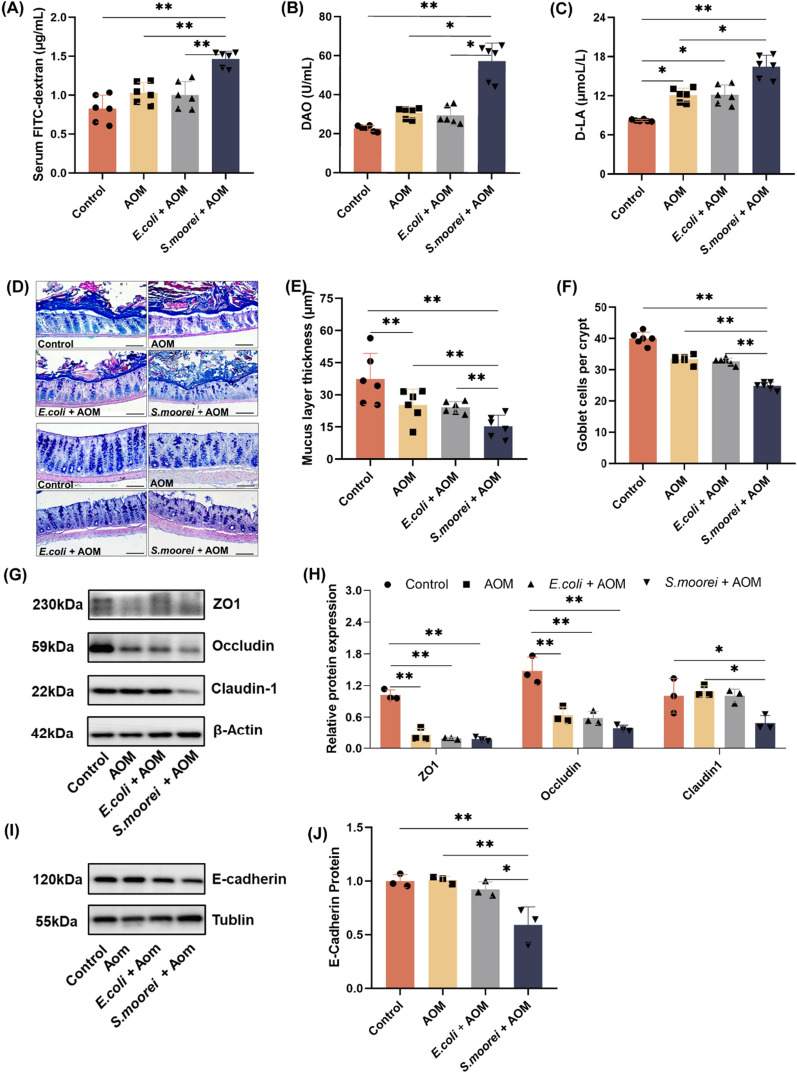
Effects of *S. moorei* on intestinal barrier function in Aom-treated mice. **A** FITC-dextran concentration in mice serum (n = 6). **B** DAO activity and **C** D-LA concentration in mice serum (n = 6). **D** Alcian blue-periodic acid Schiff staining of the colon mucosal layer and goblet cells of mice. Scale bars = 100 μm. **E** Quantitative analysis of mucosal layer thickness (n = 6). **F** Quantitative analysis of the number of goblet cells (n = 6). **J** Representative western blot images of ZO1, occludin, and claudin-1 in mice colon. **H** Quantification of ZO1, occludin, and claudin-1 protein levels (n = 3). **I** Representative western blot images of E-cadherin. **J** Quantification of E-cadherin protein levels (n = 3). *p < 0.05 and **p < 0.01 (one-way ANOVA)

To determine whether *S. moorei* impacts intestinal barrier function, several parameters including the thickness of the mucosal layer, number of goblet cells numbers, expression of tight junction proteins and adherent junction proteins in the mice colon were assessed. As shown in Fig. 5A–C, fluorescence of FITC-dextran, DAO activity, and serum levels of D-LA were significantly higher in the *S*. *moorei* group than the control and AOM groups, indicating that *S*. *moorei* induced disruption of intestinal integrity and increased permeability. As shown in Fig. 5D–F, the mucosal layer thickness and number of goblet cells in the intestinal epithelium was decreased in the AOM group as compared to the control group, while there was no significant difference between the *E. coli* and AOM groups. Collectively, these results demonstrate that *S. moorei* could disrupt the intestinal barrier of mice, thereby increasing intestinal permeability.

As compared to the control group, the protein expression levels of ZO1 and occludin were decreased by 74.2% (*p* < 0.01) and 56.7% (*p* < 0.01), respectively, in the AOM group, while there was no significant change to claudin-1 levels (Fig. 5G, H). Meanwhile, protein levels of ZO1, occludin, and claudin-1 were significantly decreased in the *S*. *moorei* group by 82.6% (*p* < 0.01), 73.7% (*p* < 0.01), and 51.3% (*p* < 0.05), respectively, as compared to the control group. These results were in agreement with the mRNA levels (Additional file 1: Fig. S7). ZO1, occludin, and claudin-1 protein levels were decreased in the *S*. *moorei* group as compared to the AOM group, but only the difference in claudin-1 protein levels were statistically significant (*p* < 0.05), while there was no significant difference between the *E. coli* and AOM groups. Similarly, E-cadherin protein levels were significantly decreased in the* S*. *moorei* group as compared to the control and AOM groups (Fig. 5I, J). Taken together, these results demonstrate that *S. moorei* may have exacerbated disease susceptibility in mice by disrupting intestinal barrier function.

### *S. moorei* promotes the production of pro-inflammatory factors by producing H_2_S and activating the NF-kB signaling pathway

**Fig. 6:**
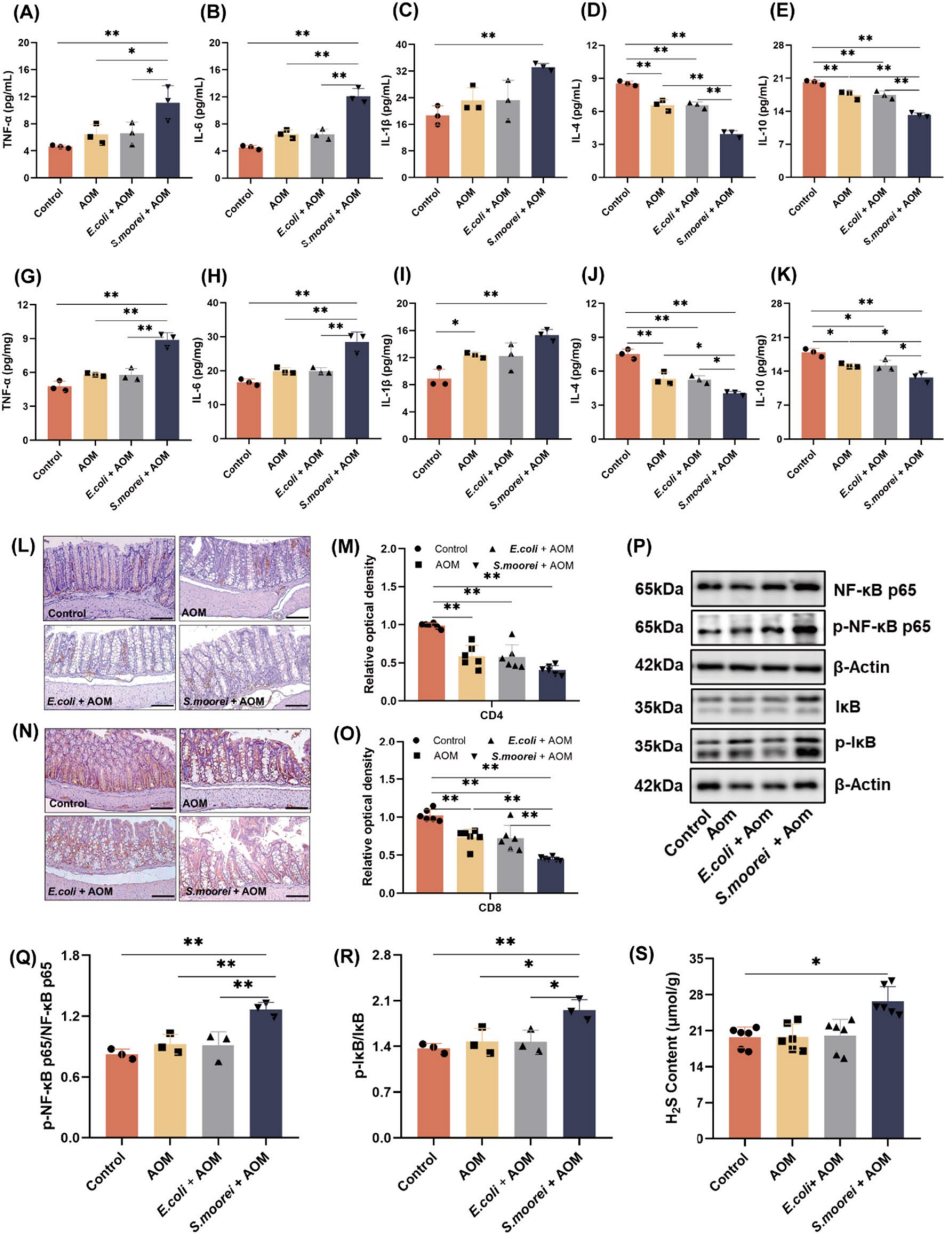
Effects of *S. moorei* on production of inflammatory factors in AOM-treated mice. **A**–**E** Concentrations of TNF-α, IL-6, IL-1β, IL-4 and IL-10 in mice serum. **G**–**K** Concentration of TNF-α, IL-6, IL-1β, IL-4 and IL-10 in mice colon tissues. **L** Representative immunohistochemical staining of CD4^+^ cells in mouse colon tissues. Scale bars = 100 μm. **M** Relative optical density (OD) of CD4^+^ cells (n = 6). **N** Representative immunohistochemical staining CD8^+^ cells in mouse colon tissues Scale bars = 100 μm. **O** Relative OD of CD8.^+^ cells (n = 6). **P** Representative western blot images of NF-κB, p-NF-κB, IκB, p-IκB in mice colon. **Q** Quantification of p-NF-κB/NF-κB levels (n = 3). **I** Quantification of p-IκB/IκB levels (n = 3). **S** H_2_S content in colon tissues of mice (n = 6). Data are presented as the mean ± SD. *P* values were calculated using one-way ANOVA. *p < 0.05, **p < 0.01

Concentrations of pro-inflammatory factors (TNF-α, IL-6, IL-1β) and anti-inflammatory factors (IL-4, IL-10) were measured in mouse serum (Fig. 6A–E) and colon tissues (Fig. 6G–K) to assess inflammatory responses. The results showed that the expression levels of all three pro-inflammatory factors were significantly increased in both serum and colon tissues of mice in the *S. moorei* group as compared to the control group (*p* < 0.01), and TNF-α and IL-6 levels were significantly increased in the serum and tissues as compared to the AOM group. In colon tissues (Fig.S8), the mRNA expression levels of TNF-α and IL-1β were significantly higher in the *S. moorei* group than the control group (*p* < 0.01). In addition, the anti-inflammatory factors IL-4 and IL-10 were significantly reduced both in the serum and colon tissues of mice in the *S. moorei* group as compared to the AOM group.

CD4^+^ and CD8^+^ cells are important components of the immune response in the intestinal tract. To further investigate the effect of *S. moorei* on inflammation in mice, immunohistochemical staining was used to detect alterations to the proportions of CD4^+^ and CD8^+^ cells in the colon tissues of mice. As shown in Fig. 6L–M, the proportion of CD4^+^ cells were significantly reduced in the AOM group as compared to the control group (*p* < 0.01). As compared to the AOM group, exposure to *S. moorei* reduced the proportion of CD4^+^ cells, although this difference was not statistically significant. Changes to the proportions of CD8^+^ cells in the mouse colon among the groups are shown in Fig. 6N–O. As compared to the control group, AOM treatment significantly reduced the proportion of CD8^+^ cells (*p* < 0.01). The proportion of CD8^+^ cells was significantly reduced in the *S. moorei* group as compared to the AOM group (*p* < 0.01). These results suggest that *S. moorei* may promote polypogenesis by suppressing proliferation of CD4^+^and CD8^+^ cells.

Previous studies have shown that H_2_S disrupts the intestinal barrier and induces production of pro-inflammatory factors, thereby promoting progression of CRC. In addition, *S. moorei* is reported to cause halitosis via production of H_2_S. Therefore, H_2_S produced by *S. moorei* could promote the development of colorectal polyps. Therefore, changes to the expression levels of proteins associated with the NF-κB signaling pathway, as well as the concentration of H_2_S in tissues, were assessed. As shown in Fig. 6P, the expression levels of the NF-kB-related proteins p-NF-κB65, NF-κB65, and p-IκB/IκB were significantly upregulated in the *S*. *moorei* group, which imply activation of the NF-kB signaling pathway (Fig. 6Q–R). As shown in Fig. 6S, the H_2_S content was significantly higher in the *S. moorei* group than the control group. Together, these results suggest that exposure to *S. moorei* may increase the expression levels of pro-inflammatory factors by inhibiting proliferation of CD4^+^ and CD8^+^ cells and activation of the NF-kB signaling pathway.

## Discussion

APs are a precursor to CRC. Growing evidence suggests that the progression of APs to CRC is related to the composition of the microbiome and chronic inflammation, which increase epithelial permeability, produce reactive oxygen species, and damage DNA [26]. Various ecological studies have focused on the identification and characterization of bacteria in health and disease [26–29]. However, the specific inflammation-related bacteria associated with progression of APs remain unclear. Inflammation induced by dysbiosis of the gut bacteria frequently involves activation of pro-inflammatory signaling pathways, as well as disruption of the gut barrier. Therefore, the focus of the present study was the roles of the characteristic bacterium *S. moorei* in progression of APs and effects on the intestinal barrier, intestinal inflammation, and proportions of immune cells in mice.

Notably, *S. moorei* was significantly enriched in the AP tissues and fecal samples, and positively associated with inflammation-related markers, suggesting that increased intestinal permeability and infiltration of inflammatory factors probably provide opportunities for colonization of *S. moorei*. *Solobacterium* is classified in the family *Erysipelotrichaceae* within the phylum Firmicutes and comprises only one unique validated species, namely *S. moorei*, which is a Gram-positive anaerobic commensal bacterium of the oral and gut microbiota [30, 31]*.* Moreover, *S. moorei*, which produces H_2_S and appears to alter the composition of oral bacteria, is reportedly enriched in the stool of patients with low-grade CRC [32]. The oral microbiome is closely related to the development of CRC [33]. Recent studies have shown that oral bacteria (e.g., *Porphyromonas gingivalis, Fusobacterium nucleatum,* and *Streptococcus*) can invade human epithelial cells and promote the progression of CRC [34]. These bacteria can also disrupt intestinal barrier function, promote production of pro-inflammatory cytokines, and increase gut permeability [35]. *S. moorei* is also reportedly involved in the pathogenesis of periodontitis, gingivitis, and blood stream infections [36–39]. In addition, the abundance of *S. moorei* was reportedly significantly higher in the feces of patients with high-grade APs than in normal controls [40]. However, it is unclear whether *S. moorei* promotes the development of APs.

In vitro studies we found that *S. moorei* more readily adhered to both CRC HT-29 cells and normal NCM460 cells than the non-pathogenic *E. coli* controls. Cell adhesion is often the first step in the pathogenesis of bacterial infections. For example, *Peptostreptococcus anaerobius* can rapidly adhere to CRC cells [41]. In the present study, exposure to *S. moorei* increased cell proliferation and production of pro-inflammatory factors and decreased production of anti-inflammatory factors as compared to the controls. A mouse model of AOM-induced APs was selected to investigate the role of *S*. *moorei *in vivo. AOM and its precursor 1,2-dimethylhydrazine are commonly used to induce dysplasia during the progression to colon cancer [42]. In general, CRC occurs in a stepwise fashion beginning with abnormal cell proliferation and dysplasia, leading to the development of APs, which is widely considered a precursor to CRC [43]. Staining for Ki67 and proteins associated with the Wnt signaling pathway confirmed *S*. *moorei* infection and hyperproliferation of colon cells. The Wnt-β-catenin signaling pathway, which is frequently activated in CRC, can be augmented by pathogenic bacteria [44, 45]. For instance, the FadA protein of *F*. *nucleatum* binds to E-cadherin of colon epithelial cells and activates β-catenin signaling, leading to increased expression of cyclin D1 and Chk2, thereby promoting tumorigenesis [46, 47]. Taken together, these results demonstrate that *S. moorei* could accelerate the development of APs in mice.

The occurrence of CRC is associated with the breakdown of the intestinal barrier. The thickness of the mucosal layer and number of goblet cells and tight junctions are often used to evaluate intestinal barrier function [48, 49]. In the present study, the thickness of the mucosal layer and the number of goblet cells were decreased in the AOM group, which were exacerbated by treatment with *S. moorei*. After AOM treatment, destruction of the mucosal layer facilitated invasion of *S. moorei*, which can reportedly decompose mucus and produce H_2_S, resulting in mucus disruption and inflammation, which contribute to the development of CRC [50]. The tight junction proteins ZO1, occludin, and claudin-1, and the adhesion junction protein E-cadherin play vital roles in maintaining cell-to-cell integrity. Thus, the loss of these proteins has been associated with the pathophysiology of a variety of gastrointestinal disorders, such as inflammatory bowel disease syndrome and even CRC. In the present study, protein expression levels of ZO1, occludin, claudin-1, and E-cadherin were significantly decreased in the *S. moorei* group as compared to the control group. As compared to the AOM group, *S. moorei* treatment significantly reduced the protein expression levels of claudin-1 and E-cadherin. Moreover, occludin protein expression was downregulated in the colorectal tissues of patients with ulcerative colitis (UC) and Crohn's disease, as was claudin-1 protein expression in the colon of patients with UC [51]. These results indicate that *S. moorei* aggravates damage to the intestinal barrier.

Chronic inflammation is an established risk factor for CRC and many pro-inflammatory mediators are positively associated with the incidence of colorectal adenoma. For example, the pro-inflammatory factors TNF-α, IL-6, and IL-1β promote tumor growth, invasion, and metastasis [52]. Serum TNF-α, IL-6, and IL-1β levels have also been associated with the poor prognosis of CRC [53]. As compared to healthy controls, CRC patients have higher serum levels of IL-6, which can activate genes associated with the proliferation of CRC cells [54]. In addition, IL-4 and IL-10, as anti-inflammatory factors, are important for maintaining gastrointestinal homeostasis and reducing the risk of CRC [55–58]. In the present study, serum levels of pro-inflammatory factors were increased, while those of anti-inflammatory factors were decreased in AP patients. Also, *S. moorei* elicited inflammatory responses both in vivo and in vitro. CD4^+^ and CD8^+^ cells play important roles in cancer immunomodulatory and immunosurveillance [56]. Therefore, the effects of *S. moorei* on the proportions of CD4^+^ and CD8^+^ cells were investigated. Previous studies have reported that *Faecalibacterium prausnitzii*, a beneficial bacterium in the gut, can prevent intestinal inflammation by inducing proliferation of CD4^+^ and CD8^+^ cells [59, 60]. The results of the present suggest that *S. moorei* induced intestinal inflammation and inhibited proliferation of CD4^+^ and CD8^+^ cells. The NF-kB signaling pathway is involved in the production of proinflammatory factors. *S. moorei*, as an oral pathogen, can produce H_2_S, a substance that causes bad breath. H_2_S production is associated with activation of the NF-kB signaling pathway. In addition, up-regulation of p-NF-kB-p65 is reported to reduce expression of tight and adhesion junction proteins [61]. Meanwhile, decreased expression of E-cadherin causes the accumulation of β-catenin, which promotes activation of the Wnt signaling pathway and progression of APs [47].

## Conclusion

In summary, the characteristic bacteria *S. moorei* in AP tissues was correlated with inflammation-related markers. Colonization of AP tissues by *S. moorei* resulted in inflammasome activation, damage to the intestinal barrier, and suppressed proliferation of CD4^+^ and CD8^+^ cells, which ultimately promotes progression of APs. Hence, targeted reduction in *S. moorei* populations in the oral cavity and gut could reduce tumor development and progression. Nevertheless, there were some limitations to this study. For example, although the role of *S. moorei* in the progression of APs was established, the underlying pathogenic mechanisms remain unclear. Thus, further investigations of probiotic or antibiotic treatments are warranted to verify the pathogenesis of *S. moorei*.

### Supplementary Information

**Additional file 1: Figure S1.** Venn diagram showing the number of OTUs in normal mucosa (C) and AP tissues (P). **Figure S2.** Wilcoxon rank-sum test comparing the flora characteristics of normal mucosa (C) and AP tissues (P) at the genus level. **Figure S3. **Composition of fecal bacteria in healthy controls and AP patients. **Figure S4. **FISH analysis of colon sections using probes against *S*. *moorei* (green), mucin2 (red), and DAPI (blue). **Figure S5.** The content of S.moorei in stool samples of S.moorei group mice. **Figure S6.** Schematic illustration of an AP in the mouse intestinal tract. **Figure S7.** Quantification of ZO1, occludin, and claudin-1 mRNA levels. **Figure S8.** TNF-α, IL-6, and IL-1β mRNA expression levels in mouse colon tissues. **Table S1.** Alpha-diversity index of microbiota in normal mucosa and AP tissues. **Table S2.** Details of colon dysplasia in mice.

## Data Availability

All data generated or analyzed in this study are included in this published article and the additional files.

## Abbreviations

Aps: Adenomatous polyps
AOM: Azoxymethane
CRC: Colorectal cancer
CRP: C-reactive protein
TNF-α: Tumor necrosis factor-α
IL: Interleukin
RIPA: Radio immunoprecipitation assay
qRT-PCR: Quantitative real-time polymerase chain reaction
OTU: Operational taxonomic unit
PCoA: Principal coordinates analysis

## References

[1] Sung H, Ferlay J, Siegel RL (2021). Global Cancer Statistics 2020: GLOBOCAN estimates of incidence and mortality worldwide for 36 cancers in 185 countries. CA Cancer J Clin.

[2] Haghighat S, Sussman DA, Deshpande A (2021). US preventive services task force recommendation statement on screening for colorectal cancer. JAMA.

[3] Strum WB (2016). Colorectal adenomas. N Engl J Med.

[4] Thibault R, Blachier F, Darcy-Vrillon B (2010). Butyrate utilization by the colonic mucosa in inflammatory bowel diseases: a transport deficiency. Inflamm Bowel Dis.

[5] Zhong X, Wang Y, Xu J (2022). Gut microbiota signatures in tissues of the colorectal polyp and normal colorectal mucosa, and faeces. Front Cell Infect Microbiol.

[6] David LA, Maurice CF, Carmody RN (2014). Diet rapidly and reproducibly alters the human gut microbiome. Nature.

[7] Zhang X, Yu D, Wu D (2023). Tissue-resident Lachnospiraceae family bacteria protect against colorectal carcinogenesis by promoting tumor immune surveillance. Cell Host Microbe..

[8] Chung L, Orberg ET, Geis AL (2018). Bacteroides fragilis toxin coordinates a pro-carcinogenic inflammatory cascade via targeting of colonic epithelial cells. Cell Host Microbe.

[9] Chai X, Wang J, Li H (2023). Intratumor microbiome features reveal antitumor potentials of intrahepatic cholangiocarcinoma. Gut Microbes.

[10] Bullman S, Pedamallu CS, Sicinska E (2017). Analysis of Fusobacterium persistence and antibiotic response in colorectal cancer. Science.

[11] Chen H, Tong T, Lu SY (2023). Urea cycle activation triggered by host-microbiota maladaptation driving colorectal tumorigenesis. Cell Metab.

[12] Shen XJ, Rawls JF, Randall T (2010). Molecular characterization of mucosal adherent bacteria and associations with colorectal adenomas. Gut Microbes.

[13] Brim H, Yooseph S, Zoetendal EG (2013). Microbiome analysis of stool samples from African Americans with colon polyps. PLoS ONE.

[14] Chen HM, Yu YN, Wang JL (2013). Decreased dietary fiber intake and structural alteration of gut microbiota in patients with advanced colorectal adenoma. Am J Clin Nutr.

[15] Lu Y, Chen J, Zheng J (2016). Mucosal adherent bacterial dysbiosis in patients with colorectal adenomas. Sci Rep.

[16] Mangifesta M, Mancabelli L, Milani C (2018). Mucosal microbiota of intestinal polyps reveals putative biomarkers of colorectal cancer. Sci Rep.

[17] Yu S, Wang J, Li Y, Wang X (2021). Structural studies of water-insoluble beta-glucan from oat bran and its effect on improving lipid metabolism in mice fed high-fat diet. Nutrients.

[18] Ren Y, Yu G, Shi C (2022). Majorbio cloud: a one-stop, comprehensive bioinformatic platform for multiomics analyses. iMeta.

[19] Magoc T, Salzberg SL (2011). FLASH: fast length adjustment of short reads to improve genome assemblies. Bioinformatics.

[20] Edgar RC (2013). UPARSE: highly accurate OTU sequences from microbial amplicon reads. Nat Methods.

[21] Wang Q, Garrity GM, Tiedje JM (2007). Naive bayesian classifier for rapid assignment of rRNA sequences into the new bacterial taxonomy. Appl Environ Microb.

[22] Langmead B, Salzberg SL (2012). Fast gapped-read alignment with Bowtie 2. Nat Methods.

[23] Bolger AM, Lohse M, Usadel B (2014). Trimmomatic: a flexible trimmer for Illumina sequence data. Bioinformatics.

[24] Wong SH, Zhao L, Zhang X (2017). Gavage of fecal samples from patients with colorectal cancer promotes intestinal carcinogenesis in germ-free and conventional mice. Gastroenterology.

[25] Kang X, Ng S-K, Liu C (2023). Altered gut microbiota of obesity subjects promotes colorectal carcinogenesis in mice. eBioMedicine.

[26] Xue X, Li R, Chen Z (2023). The role of the symbiotic microecosystem in cancer: gut microbiota, metabolome, and host immunome. Front Immunol.

[27] Camp JG, Kanther M, Semova I (2009). Patterns and scales in gastrointestinal microbial ecology. Gastroenterology.

[28] Eckburg PB, Bik EM, Bernstein CN (2005). Diversity of the human intestinal microbial flora. Science.

[29] Palmer C, Bik EM, DiGiulio DB (2007). Development of the human infant intestinal microbiota. PLoS Biol.

[30] Avuthu N, Guda C (2022). Meta-analysis of altered gut microbiota reveals microbial and metabolic biomarkers for colorectal cancer. Microbiol Spectr.

[31] Alauzet C, Aujoulat F, Lozniewski A (2021). A new look at the genus Solobacterium: a retrospective analysis of twenty-seven cases of infection involving S moorei and a review of sequence databases and the literature. Microorganisms.

[32] Yu J, Feng Q, Wong SH (2017). Metagenomic analysis of faecal microbiome as a tool towards targeted non-invasive biomarkers for colorectal cancer. Gut.

[33] Flemer B, Warren RD, Barrett MP (2018). The oral microbiota in colorectal cancer is distinctive and predictive. Gut.

[34] Younginger BS, Mayba O, Reeder J (2023). Enrichment of oral-derived bacteria in inflamed colorectal tumors and distinct associations of Fusobacterium in the mesenchymal subtype. Cell Rep Med.

[35] Louis P, Hold GL, Flint HJ (2014). The gut microbiota, bacterial metabolites and colorectal cancer. Nat Rev Microbiol.

[36] Yu Q, Xu Q, Zhu YJ (2023). Bloodstream infection caused by Solobacterium moorei: a case report and literature review. Indian J Med Microbiol.

[37] Vancauwenberghe F, Dadamio J, Laleman I (2013). The role of Solobacterium moorei in oral malodour. J Breath Res.

[38] Morin MP, Bedran TB, Fournier-Larente J (2015). Green tea extract and its major constituent epigallocatechin-3-gallate inhibit growth and halitosis-related properties of Solobacterium moorei. BMC Complement Altern Med.

[39] Barrak I, Stajer A, Gajdacs M (2020). Small, but smelly: the importance of Solobacterium moorei in halitosis and other human infections. Heliyon.

[40] Yachida S, Mizutani S, Shiroma H, Shiba S, Nakajima T, Sakamoto T (2019). Metagenomic and metabolomic analyses reveal distinct stage-specific phenotypes of the gut microbiota in colorectal cancer. Nat Med.

[41] Tsoi H, Chu ESH, Zhang X (2017). Peptostreptococcus anaerobius induces intracellular cholesterol biosynthesis in colon cells to induce proliferation and causes dysplasia in mice. Gastroenterology.

[42] Perse M, Cerar A (2011). Morphological and molecular alterations in 1,2 dimethylhydrazine and azoxymethane induced colon carcinogenesis in rats. J Biomed Biotechnol.

[43] Morson BC (1974). Evolution of cancer of the colon and rectum. Cancer.

[44] Drewes JL, Chen J, Markham NO (2022). Human colon cancer-derived clostridioides difficile strains drive colonic tumorigenesis in mice. Cancer Discov.

[45] Li L, Li X, Zhong W (2019). Gut microbiota from colorectal cancer patients enhances the progression of intestinal adenoma in Apcmin/+ mice. EBioMedicine.

[46] Wang Q, Lin Y, Sheng X (2020). Arachidonic acid promotes intestinal regeneration by activating wnt signaling. Stem Cell Rep.

[47] Rubinstein MR, Wang X, Liu W (2013). Fusobacterium nucleatum promotes colorectal carcinogenesis by modulating E-cadherin/β-catenin signaling via its FadA adhesin. Cell Host Microbe.

[48] Johansson ME, Sjovall H, Hansson GC (2013). The gastrointestinal mucus system in health and disease. Nat Rev Gastroenterol Hepatol.

[49] Swank GM, Deitch EA (1996). Role of the gut in multiple organ failure: bacterial translocation and permeability changes. World J Surg.

[50] Buret AG, Allain T, Motta JP (2022). Effects of hydrogen sulfide on the microbiome: from toxicity to therapy. Antioxid Redox Signal.

[51] Martin TA, Jiang WG (2009). Loss of tight junction barrier function and its role in cancer metastasis. Biochim Biophys Acta.

[52] Ray AL, Berggren KL, Restrepo Cruz S (2018). Inhibition of MK2 suppresses IL-1beta, IL-6, and TNF-alpha-dependent colorectal cancer growth. Int J Cancer.

[53] Chang PH, Pan YP, Fan CW (2016). Pretreatment serum interleukin-1beta, interleukin-6, and tumor necrosis factor-alpha levels predict the progression of colorectal cancer. Cancer Med.

[54] Song M, Mehta RS, Wu K (2016). Plasma inflammatory markers and risk of advanced colorectal adenoma in women. Cancer Prev Res (Phila).

[55] Avisar A, Cohen M, Aharon A, Katz R, Bar-Sela G (2023). Positive affect and fatigue as predictors of anti-inflammatory IL-10 concentrations among colorectal cancer patients during adjuvant chemotherapy. J Psychosom Res.

[56] Rizzo A, Pallone F, Monteleone G, Fantini MC (2011). Intestinal inflammation and colorectal cancer: a double-edged sword?. World J Gastroenterol.

[57] Formentini A, Braun P, Fricke H, Link KH, Henne-Bruns D, Kornmann M (2012). Expression of interleukin-4 and interleukin-13 and their receptors in colorectal cancer. Int J Colorectal Dis.

[58] Mannino MH, Zhu Z, Xiao H, Bai Q, Wakefield MR, Fang Y (2015). The paradoxical role of IL-10 in immunity and cancer. Cancer Lett.

[59] Touch S, Godefroy E, Rolhion N, Danne C, Oeuvray C, Straube M, Galbert C, Brot L, Alonso Salgueiro I, Chadi S, Ledent T, Chatel JM, Langella P, Jotereau F, Altare F, Sokol H (2022). Human CD4+CD8α+ Tregs induced by Faecalibacterium prausnitzii protect against intestinal inflammation. JCI Insight.

[60] Alameddine J, Godefroy E, Papargyris L, Sarrabayrouse G, Tabiasco J, Bridonneau C (2019). Faecalibacterium prausnitzii skews human DC to prime IL10-producing T cells through TLR2/6/JNK signaling and IL-10, IL-27, CD39, and IDO-1 induction. Front Immunol.

[61] Drolia R, Tenguria S, Durkes AC (2018). Listeria adhesion protein induces intestinal epithelial barrier dysfunction for bacterial translocation. Cell Host Microbe.

