# Supplementary material for: Solobacterium moorei promotes the progression of adenomatous polyps by causing inflammation and disrupting the intestinal barrier
Source: J Transl Med. 2024 Feb 17;22:169. doi: 10.1186/s12967-024-04977-3 (PMC10874563; doi:10.1186/s12967-024-04977-3)
Supplement: Supplementary file 1 — Additional file 1: Figure S1. Venn diagram showing the number of OTUs in normal mucosa (C) and AP tissues (P). Figure S2. Wilcoxon rank-sum test comparing the flora characteristics of normal mucosa (C) and AP tissues (P) at the genus level. Figure S3. Composition of fecal bacteria in healthy controls and AP patients. Figure S4. FISH analysis of colon sections using probes against S. moorei (green), mucin2 (red), and DAPI (blue). Figure S5. The content of S.moorei in stool samples of S.moorei group mice. Figure S6. Schematic illustration of an AP in the mouse intestinal tract. Figure S7. Quantification of ZO1, occludin, and claudin-1 mRNA levels. Figure S8. TNF-α, IL-6, and IL-1β mRNA expression levels in mouse colon tissues. Table S1. Alpha-diversity index of microbiota in normal mucosa and AP tissues. Table S2. Details of colon dysplasia in mice. [file 12967_2024_4977_MOESM1_ESM.docx]

**Supplementary material**


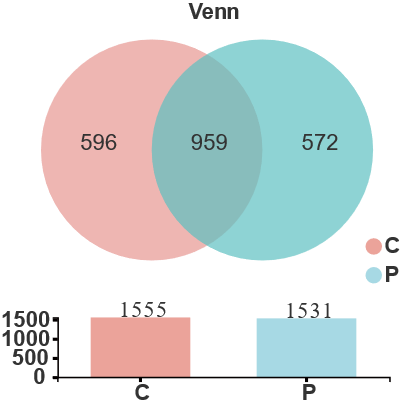


**Figure S1** **Venn diagram showing the number of OTUs in normal mucosa (C) and AP tissues (P)**

**
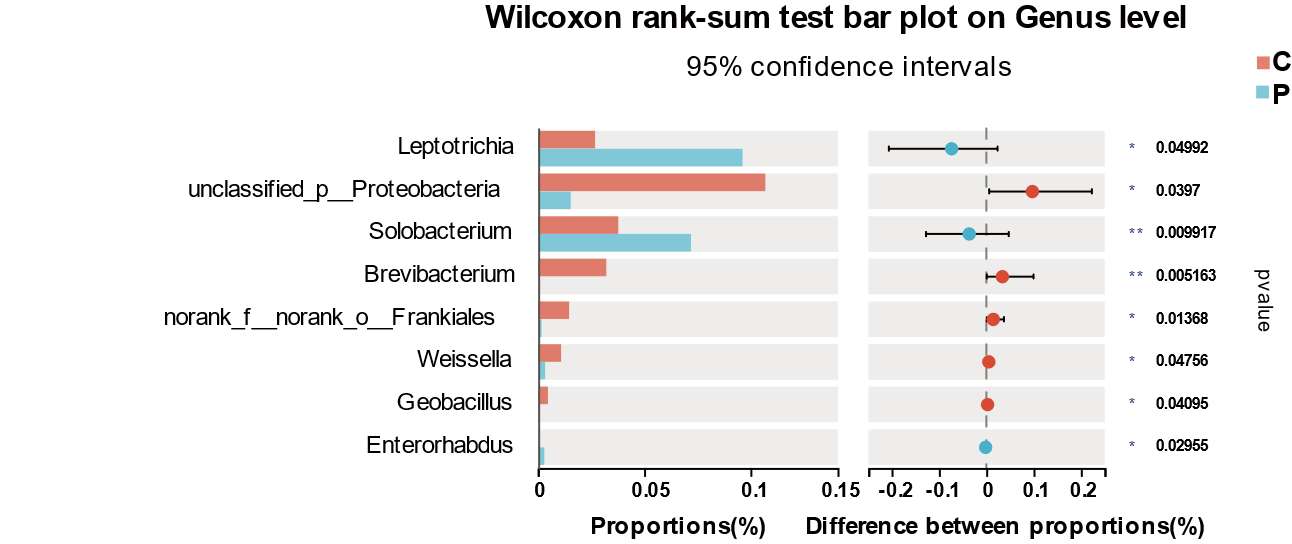
**

**Figure S2 Wilcoxon rank-sum test comparing the flora characteristics of normal mucosa (C) and AP tissues (P) at the genus level.**


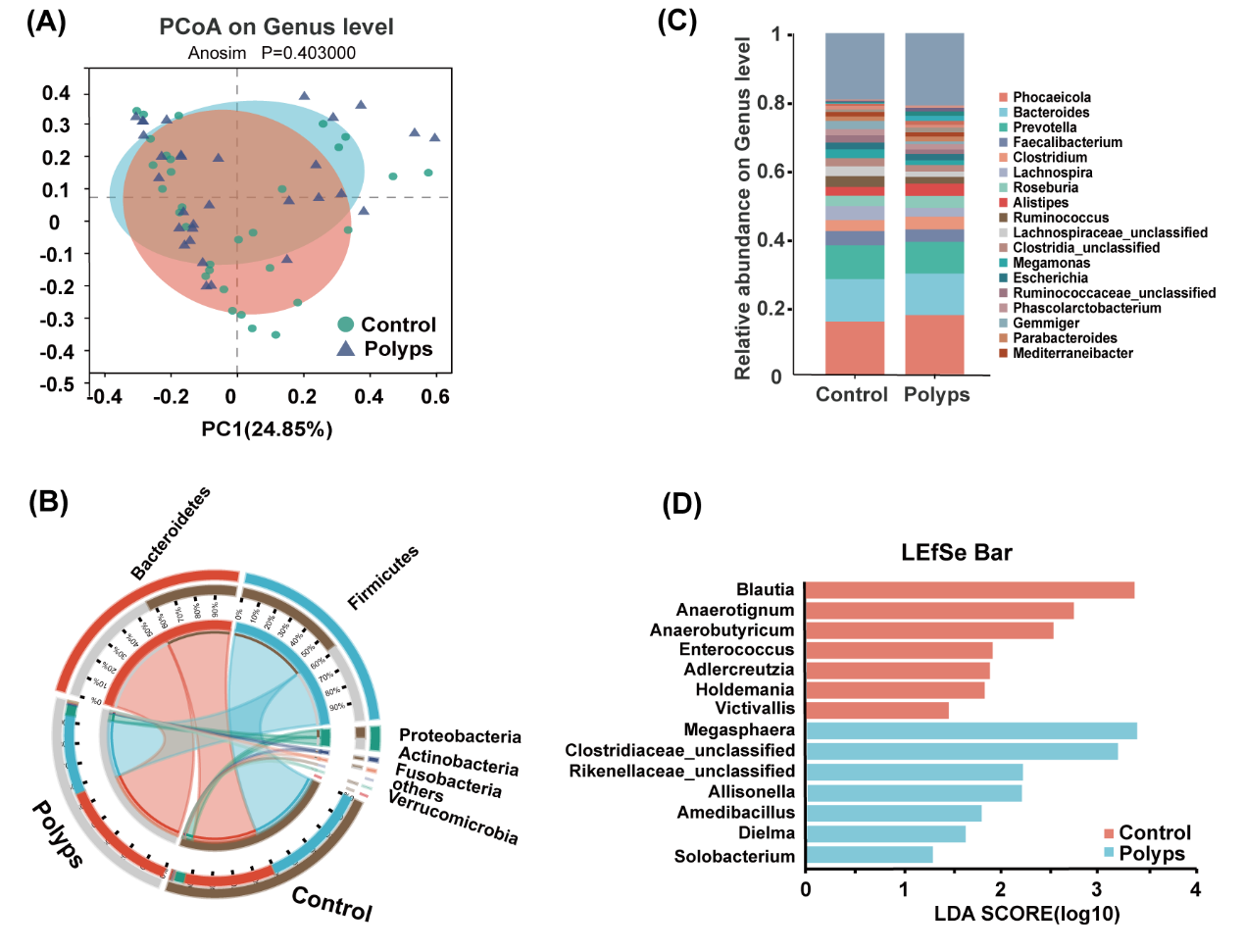


**Figure S3** **Composition of fecal bacteria in healthy controls and** **AP patients.**

(A) Principal coordinate analysis (PCoA) plot representing unweighted Unifrac distances. *p*-values were calculated using anosim.

(B) The relative abundance of phylum in healthy controls and colorectal polyps.

(C) The relative abundance of genus in healthy controls and colorectal polyps.

(D) LEfSe analysis. The criteria for feature selection were log LDA score >1.2.

Control: healthy controls, Polyps: AP patients.

**
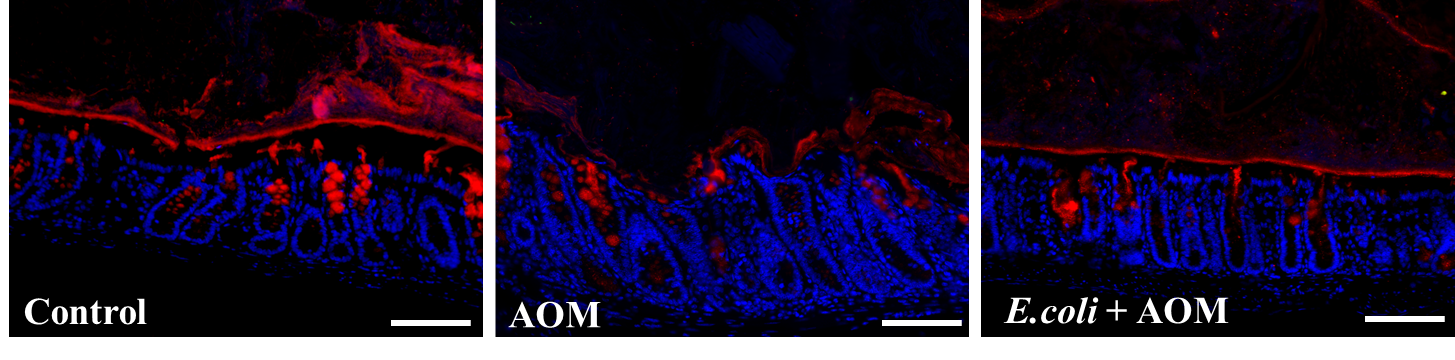
**

**Figure S4** **FISH analysis of colon sections using probes against S. moorei (green), mucin2 (red), and DAPI (blue).**


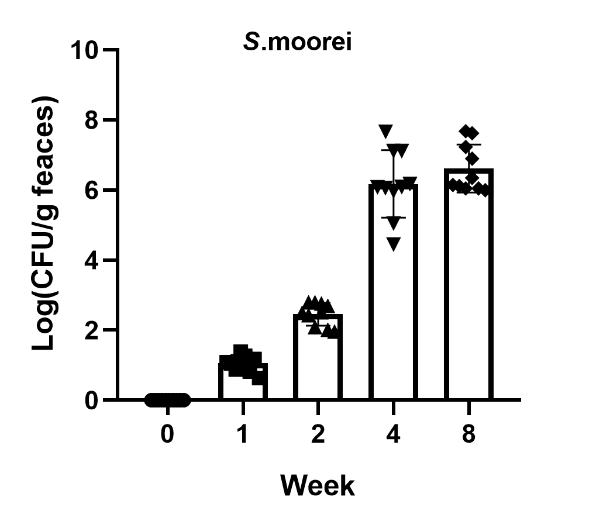


**Figure S5 The content of *S.moorei* in stool samples of *S.moorei* group mice.**


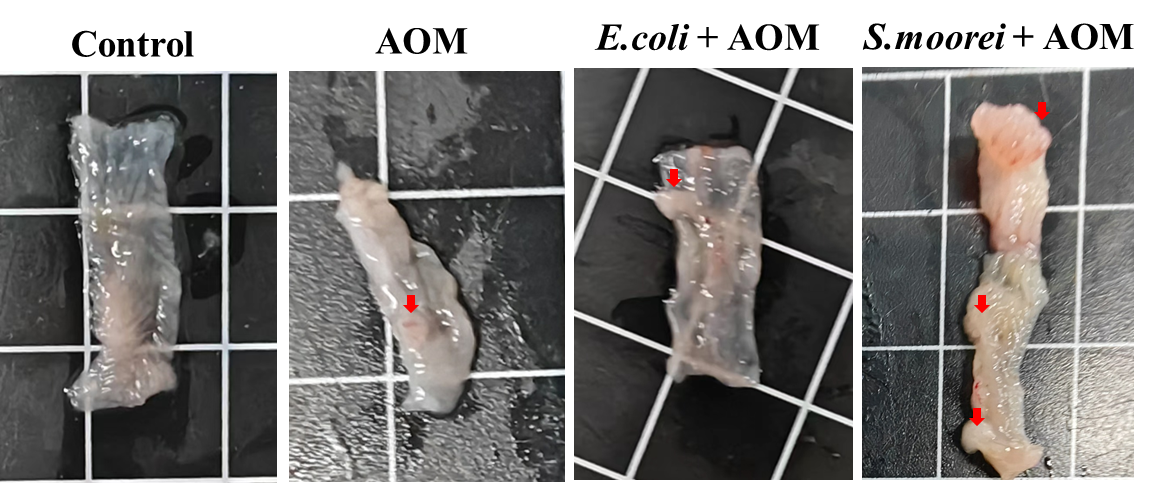


**Figure S6** **Schematic illustration of an AP in the mouse intestinal tract.**

**
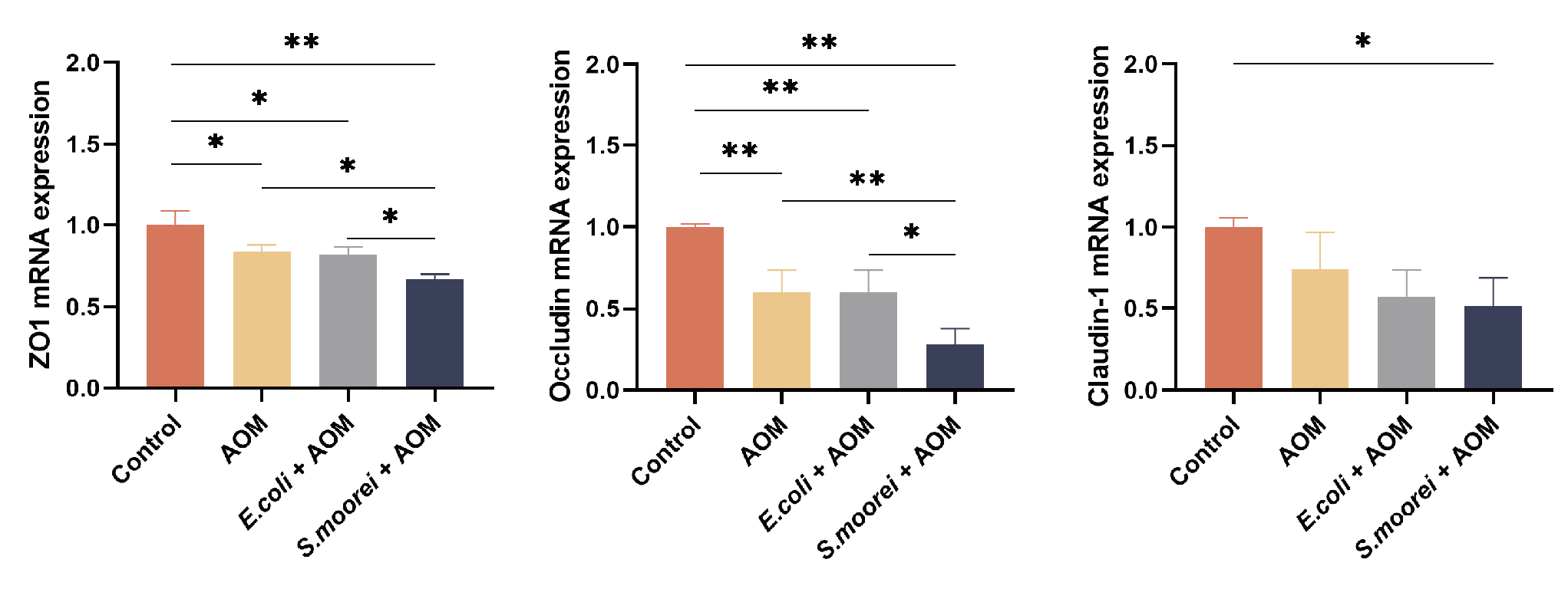
**

**Figure S7 Quantification of ZO1, occludin, and claudin-1 mRNA levels.**

**
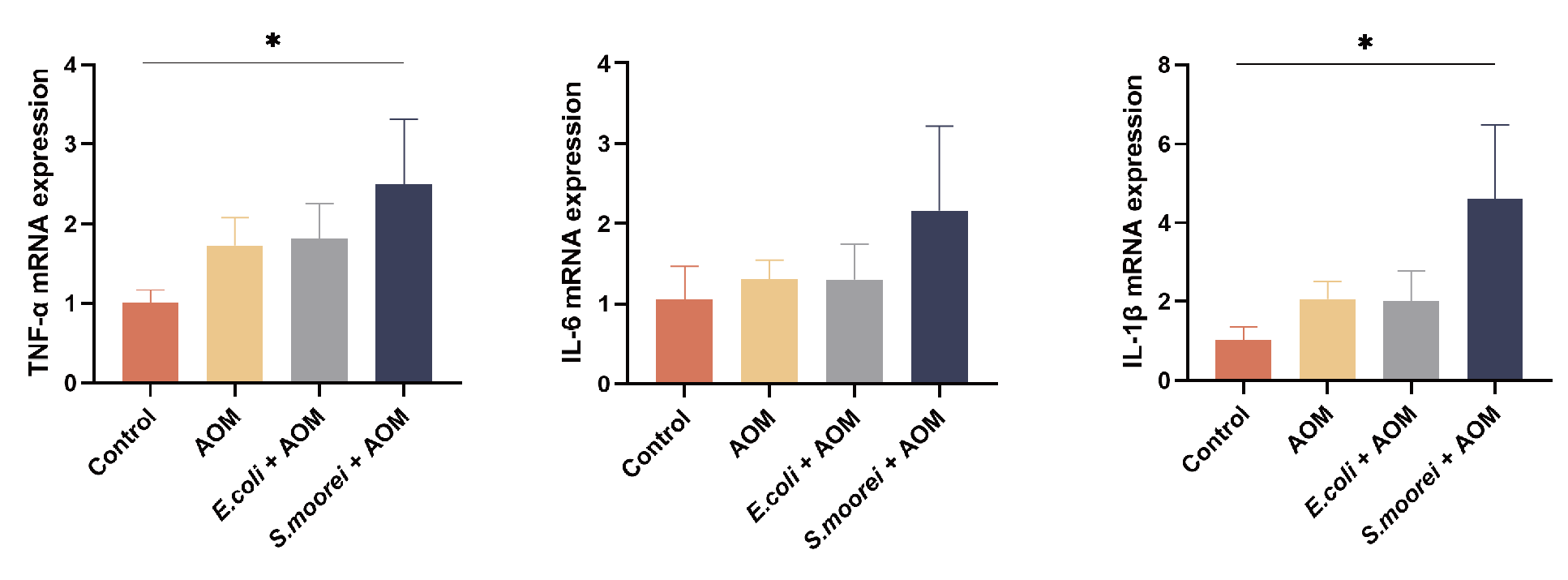
**

**Figure S8** **TNF-α, IL-6, and IL-1β mRNA expression levels in mouse colon tissues.**

**Table S1 Alpha-Diversity index of microbiota in the normal mucosa and AP tissues**

| **Group** | **Sobs** | **Shannon** | **Simpson** | **Ace** | **Chao** |
| --- | --- | --- | --- | --- | --- |
| C | 214.78 ± 66.73 | 2.86 ± 0.96 | 0.20 ± 0.19 | 264.94 ± 127.98 | 242.67 ± 66.57 |
| *p* | 210.86 ± 93.23 | 2.71 ± 1.21 | 0.25 ± 0.27 | 228.64 ± 95.03 | 229.53 ± 100.02 |
| *p* value | 0.89 | 0.92 | 0.89 | 0.40 | 0.61 |

Data are expressed as mean ± SD. C, normal mucosa (n=32); P, AP tissues (n=28).

**Table S2** **Details of colon dysplasia in mice**

| **Group** | **Control** | **AOM** | ***E.coli* + AOM** | ***S.moorei* + AOM** |
| --- | --- | --- | --- | --- |
| Total no. mice | 10 | 10 | 10 | 10 |
| No. of polyps, mean | 0 | 0.8 | 0.9 | 2.9 |
| Polyps size, mm, mean ± SD | 0 | 0.35 ± 0.32 | 0.37 ± 0.35 | 1.32 ± 0.96 |
| Mice with dysplasis, n (%) | 0 (0) | 2(20) | 2 (20) | 6 (60) |
| High-grade dysplasis, n (%) | 0 (0) | 0 (0) | 0 (0) | 2 (20) |
| Mice with inflammation, n (%) | 0 (0) | 4 (40) | 5(50) | 8 (80) |
| Histology score,mean ± SD | 0 | 1.30 ± 1.16 | 1.30 ± 1.06 | 2.70 ± 0.95 |
